# Comprehensive Strategies in Endodontic Pain Management: An Integrative Narrative Review

**DOI:** 10.7759/cureus.50371

**Published:** 2023-12-12

**Authors:** Ali M Falatah, Reem S Almalki, Ahmad S Al-Qahtani, Bayadir O Aljumaah, Weaam K Almihdar, Ahmed S Almutairi

**Affiliations:** 1 Dentistry, King Fahd Hospital, Madinah, SAU; 2 Dentistry, King Saud bin Abdulaziz University for Health and Sciences, Riyadh, SAU; 3 Dentistry, Ministry of Health, Riyadh, SAU; 4 General Practice, Qassim University, Qassim, SAU; 5 Dentistry, National Guard Hospital, Madinah, SAU; 6 Dentistry, Magrabi Centers and Hospitals, Al-Kharj, SAU

**Keywords:** post endodontic pain, post operative endodontic pain, endodontic, endodontic management, endodontic treatment

## Abstract

This narrative review comprehensively examines the current and emerging strategies for pain management in endodontics, encompassing a wide range of pharmacological and non-pharmacological approaches. Through an extensive exploration of 20 distinct parts, the review discusses traditional analgesics, antibiotics, the use of corticosteroids, and the role of novel treatments such as platelet-rich fibrin (PRF) and cryotherapy. The review also delves into the intricacies of clinical methods, such as trephination and occlusal reduction, and discusses the potential of advanced techniques such as GABAergic signaling, acupuncture, in silico modulation, and low-level laser therapy (LLLT) for the effective management of endodontic pain. The analysis reveals a trend toward integrative methods that combine established practices with cutting-edge research, highlighting the importance of a tailored approach in endodontic pain management. The findings underscore the significance of understanding the complex nature of dental pain and the need for multifaceted treatment strategies. The review emphasizes that while traditional pharmacological methods remain foundational, emerging therapies offer promising adjuncts or alternatives, especially in cases where conventional treatments may be inadequate or unsuitable. This review aims to serve as a comprehensive resource for endodontic practitioners and researchers, offering insights into the multifarious aspects of pain management in endodontics. It underscores the ongoing evolution in the field and suggests directions for future research, particularly in refining and validating new pain management techniques.

## Introduction and background

Webster’s dictionary defines pain as “a basic bodily sensation induced by a noxious stimulus, received by naked nerve endings, associated with actual or potential tissue damage, characterized by physical discomfort (such as pricking, throbbing, or aching), and typically leading to evasive action.” The distress originating from dental pain aligns with this definition. The dental pulp, with its complex sensory and vascular system, comprises A (myelinated) and C (unmyelinated) nerve fibers [1]. It senses local stimuli, resulting in an inflammatory response that increases intrapulpal pressure and consequently activates free nerve endings [2]. The pain perceived varies in intensity; it can be dull (through C nerve fibers), constant, sharp (through A nerve fibers) [3], or throbbing. The complexity of dental pain is further compounded by its multifactorial etiology, including dental caries (microbial), trauma, periodontitis, iatrogenic damage, or other irritants [4].

Identifying the cause of the pulpal insult and establishing a diagnosis lead to treatment decisions [4-5], which involve retaining or extracting the offending tooth. However, this process is not as straightforward as it seems, as it involves a cascade of management protocols categorized as preoperative, perioperative (intra-appointment and inter-appointment), and postoperative pain management for successful treatment outcomes [6-7]. In cases where pulpal diagnosis favors routine nonsurgical endodontic treatment (root canal treatment [RCT]), the management of endodontic pain should follow the same guidelines to provide patient-centered care. Nagendrababu et al. emphasized that prioritizing what is most significant to the patient through dentist-patient-reported outcomes (dPRO) benefits the entire practice and society [8]. RCT is associated with high levels of fear and anxiety, which exacerbate the patient’s discomfort [9]. Preoperatively, assessing the patient’s current disease status and taking measures to reduce the infectious state, if present, is crucial for pain control. Behavioral management, pharmacologic methods (nonnarcotic), and profound anesthesia are essential [7]. When necessary, dentists may opt for narcotics (opioids), considering the risk-benefit ratio [7].

Intra-appointment pain management strategies vary from case to case. For example, when local anesthesia (infiltration or inferior alveolar nerve block [IANB]) is inadequate in acute irreversible pulpitis due to inflamed tissues and low pH [7,10], clinicians may opt for different concentrations or supplemental injections. Pretreatment medication also plays a favorable role. Additionally, perioperative clinical measures such as incision and drainage, occlusal reduction, and trephination can be adjunctive [10]. Despite strict protocols and adherence to infection control guidelines, flare-ups, particularly in necrotic teeth due to microorganism involvement, are possible [11]. Postoperatively, approximately 1.4% to 1.6% of cases may result in pain, but as the study suggests, time is an essential factor. While mild-to-moderate pain often resolves over time, severe pain requires further investigation and management [11].

The review article aims to establish conventional and cutting-edge strategies for managing endodontic pain at all three levels (preoperative, perioperative, and postoperative) and the future prospects for pain suppression to yield broader benefits.

## Review

Materials and methods

For this narrative review, a comprehensive database search was conducted using platforms such as PubMed, Google Scholar, and ScienceDirect. The search employed manual keyword techniques with terms, including "pain management," "endodontic," "preoperative pain," "postoperative pain," "inter-appointment pain," "perioperative pain," "analgesia," and "anesthesia." The selection criteria focused on articles directly related to the topic or those with significant association with the subject matter. This review encompasses literature from the past, starting from 1986, up to the present time (March 2023). Additionally, articles identified through references in the initially retrieved papers were also included.

Eligibility criteria

Inclusion Criteria

The search strategy used the PICO criteria and focused on articles in English, with or without full-text abstracts. The articles included were related to pain management in endodontics, covering the preoperative, postoperative, and perioperative phases. The review also included literature that compared traditional methods with potential new methods and studies exploring future prospects in the field. Additionally, relevant studies covering pain physiology, patient apprehension, and management of emergencies or flare-ups were included. The review incorporated systematic reviews (with or without meta-analysis), related narrative reviews, randomized controlled clinical trials, and select surveys.

Exclusion Criteria

Exclusion criteria encompassed unrelated literature, case reports, unpublished data, studies not in English, and those that focused on sources of dental pain other than endodontics. Due to the comprehensive nature of the subject matter, a narrative review format was chosen over a systematic review. This methodology allowed for an extensive exploration of a wide range of topics while taking into consideration the heterogeneous nature of data. In addition, time constraints made it less feasible to conduct a systematic review. Therefore, a more flexible approach was adopted to qualitatively analyze and synthesize information on endodontic pain management.

Discussion

Clinicians have long faced the challenge of improving pain management strategies in their practice, yet effective pain management remains an ongoing endeavor [12]. While the commonly advocated 3D approach (diagnosis, definitive treatment, and drugs) is a standard in resolving endodontic emergencies [13], it does not always guarantee a successful outcome. This is particularly evident as high incidences of postoperative pain have been reported even after addressing painful conditions like irreversible pulpitis and apical periodontitis through RCT [14]. This reality underscores clinicians' need to accurately differentiate between inflammation and infection to address the root cause effectively. Consequently, the field continually pursues developing and refining methods for managing endodontic pain more effectively:

Behavioral management

Behavioral management is crucial when a patient first visits the dental office, during treatment, between appointments, and even after the procedure. This phenomenon is not new, as many patients experience apprehension related to toothache or dental procedures. In 1986, a study was conducted in Seattle, United States, to investigate dental fears, dental experiences, and perceived oral health status among 1,019 residents. The study found that around 204 per 1,000 people in Seattle were affected by high dental fear, with the majority (66%) developing this fear in their early childhood years [15]. The expectation of a painful experience can increase a patient's anxiety levels, making treatment more challenging [16]. This anxiety can lower the pain threshold, and prolonged visits can add stress for both the patient and the dentist.

Research has shown that there are several ways to enhance the overall patient experience. One approach is to create a safer environment, which can help patients feel more at ease. A recent review article titled *An Update on Pain Control in Conservative Dentistry and Endodontics* further discussed the importance of communicating effectively with patients, familiarizing them with the procedure, and using distraction techniques. For younger patients, its recommended techniques include adjusting the atmosphere with lavender scents, involving them in discussions, and employing rapport-building techniques such as voice control, distraction, modeling, and memory reconstruction [17].

Pharmacological management

Analgesics

Analgesics used in dental practice vary widely, including non-opioid analgesics like acetaminophen; opioid analgesics such as tramadol and codeine; nonsteroidal anti-inflammatory drugs (NSAIDs) like ibuprofen, flurbiprofen, ketorolac tromethamine, etodolac, tenoxicam, and naproxen; steroidal anti-inflammatory drugs (e.g., prednisolone); and combinations of these therapies. A clinical study titled *Efficacy of Routinely Used Analgesics in Management of Pulpal Pain Postoperatively* evaluated the effectiveness of various analgesics in pain management. The study grouped patients into five groups: Group I received aceclofenac + paracetamol, Group II took ketorolac tromethamine, Group III was given lornoxicam + paracetamol, Group IV received tramadol + paracetamol, and Group V was administered paracetamol. Patients were asked to rate their pain levels at 4, 6, 8, 24, and 48 hours posttreatment using the visual analog scale (VAS) scores. The study concluded that there was no significant difference in the efficacy of these medications for pain management. However, it was noted that Groups III and IV demonstrated benefits due to their sustained release capabilities. Ketorolac was effective for short-term pain relief. Paracetamol is recommended for immune-compromised patients where safety is a concern [18].

Non-steroidal Anti-inflammatory Drugs

NSAIDs are widely used in dental practice, particularly for their effectiveness in relieving post-endodontic pain (PEP). These drugs work by inhibiting prostaglandin synthesis, which is achieved through the decreased activity of the cyclooxygenase (Cox) 1 and Cox 2 [19]. This action is pivotal in pain management in many dental patients [20].

Diclofenac sodium: This specific NSAID functions by blocking the Cox enzymes and is commonly prescribed for dental pain and swelling postoperatively. Postoperative administration of NSAIDs like diclofenac sodium can significantly reduce a patient’s opioid requirements, thereby decreasing the occurrence and severity of opioid-induced adverse events (AEs) [21]. One study investigated the effectiveness of diclofenac sodium in pain management and compared its effectiveness when delivered orally versus via the intraligamentary route. The study found that prophylactic intraligamentary injection of diclofenac sodium was more effective than oral administration in reducing PEP [16].

Furthermore, another study emphasized that NSAIDs are the most commonly used medication for preventing and controlling postoperative pain [22].

Periapical Injection of Ketorolac

Effective pain management in endodontic procedures is crucial. Ketorolac tromethamine, the first NSAID available for intramuscular injection in the United States, is as effective as opiates when injected intramuscularly. While its intraoral efficacy has been explored in several clinical studies, more research is needed to fully understand its benefits and limitations in this application. In a study involving 52 patients undergoing endodontic treatment, the analgesic effects of various injections, including placebo/placebo, 30 mg ketorolac/placebo, placebo/30 mg ketorolac, and 2% mepivacaine with 1:20 K levonordefrin/placebo, were evaluated in a double-blind manner [23]. The results indicated that intraoral infiltration of ketorolac, particularly for mandibular pain, significantly reduced discomfort, suggesting its potential as an effective pain management method in endodontic procedures. However, the study had certain limitations. Its sample size, though offering valuable insights, might not be adequate to generalize the findings to a larger population. Additionally, the study focused mainly on the immediate analgesic effects of ketorolac, without extensively exploring aspects like possible side effects or the drug's efficacy with repeated use. Consequently, further research involving a larger and more diverse patient sample is necessary to fully establish the effectiveness and safety of ketorolac in endodontic pain management [23].

Intranasal (IN) Analgesics

Although oral administration of drugs is predominant in dental prescriptions, the advantages of IN delivery, such as enhanced absorption due to a greater surface area-to-volume ratio, are increasingly recognized. In endodontic pain management, a prospective, double-blind clinical study showed that IN ketorolac significantly reduced both preoperative and postoperative pain [24]. Another study investigated IN ketorolac's efficacy in an acute pain model, with untreated endodontic patients experiencing moderate-to-severe pain from symptomatic apical periodontitis, alongside paracetamol/ibuprofen administration [25]. This study found that IN ketorolac, while not significantly outperforming ibuprofen/paracetamol, offers a nonnarcotic alternative for drug delivery, which is a valuable option for clinicians.

However, these studies have limitations. The small sample sizes may limit the ability to generalize the findings to a broader patient population. Also, their focus on specific pain conditions might not fully capture the effectiveness of IN ketorolac in other types of dental pain. Further research with larger and more varied patient groups is essential to confirm these initial findings and to fully establish IN ketorolac as a versatile option in endodontic pain management.

*Corticosteroids*

Corticosteroids are known for their anti-inflammatory and immune-suppressive effects. Their efficiency in managing endodontic pain has been affirmed in numerous studies, with a consensus on their effectiveness as a modality for endodontic pain relief. A systematic review and meta-analysis titled *Efficacy of Corticosteroids on Postoperative Endodontic Pain: A Systematic Review and Meta-analysis* found that corticosteroids have a postoperative pain-reducing effect in endodontic patients. Interestingly, this study highlighted how different drug regimens can influence the effect size at various time points [26].

Methylprednisolone, a potent steroid against inflammation, has been widely studied in pain management. A randomized clinical trial titled *Intraosseous Methylprednisolone Injection for Acute Pulpitis Pain* compared the efficacy of a minimally invasive procedure - intraosseous methylprednisolone injection - with pulpotomy for pain relief in acute pulpitis over a seven-day observation period. The study concluded that methylprednisolone injection for acute pulpitis was more beneficial than pulpotomy after seven days, conserving scarce dental resources such as endodontic equipment and supplies and the dental surgeon's time. However, no significant difference in therapeutic outcomes was observed between the two treatment groups after six months [22].

*Trypsin-Chymotrypsin*

Trypsin-chymotrypsin, known for its anti-inflammatory and antioxidant properties, is recognized for reducing postoperative swelling and managing severe pain. A randomized control trial conducted in 2003 investigated the effect of trypsin-chymotrypsin on postoperative pain following a single visit of endodontic treatment of mandibular first molars with symptomatic irreversible pulpitis. The study divided 60 patients into four groups of fifteen each, with each group receiving either ibuprofen (600 mg), ambezim-G (trypsin 5 mg and chymotrypsin 5 mg), a combination of both, or a placebo, postoperatively. Participants’ pain levels were assessed at various intervals using a numerical scale [27].

The findings indicated no significant difference in pain relief among the ibuprofen, trypsin-chymotrypsin, and combination groups, but these groups all showed marked pain relief compared to the placebo group. Interestingly, the study also concluded that there was no synergistic effect between chymotrypsin-trypsin and NSAIDs, as their efficacy was similar. However, it's important to note the limitation of the study: the sample size of 15 patients per group may not be large enough to fully represent a broader patient population. This limitation suggests that while the results are promising, further research with a larger sample size is necessary to validate these findings and to better understand the potential benefits and limitations of trypsin-chymotrypsin in endodontic pain management [27].

Opioids/Narcotic Analgesics

The United States is currently facing an unprecedented epidemic primarily attributed to the extensive prescription of opioids. It is both intriguing and concerning to note that while dentistry is considered a minor healthcare sector, the volume of opioid prescriptions in this field significantly contributes to the overall number of such prescriptions [28]. Owing to this alarming situation, opioid prescription is typically reserved for severe cases where pain has not been adequately managed by nonnarcotic analgesics or in combination with them. The practice of prescribing opioids, while clinically supported, is complicated due to their severe adverse effects [29]. This highlights the need for comprehensive training for dentists and robust research aimed at transitioning to safer pain management options [30]. A descriptive cross-sectional survey titled *An Analysis of Current Analgesic Preferences for Endodontic Pain Management* explored this issue further. The survey presented respondents with various choices for analgesic prescriptions, including different doses of ibuprofen or acetaminophen (APAP) and combination narcotic medications. The results showed that nonnarcotic medications were preferred over narcotics in all clinical situations [31].

Prophylactic Antibiotics

Antibiotics have long been a fundamental element in dentistry. Their use and potential overuse have been extensively explored in literature. Several studies have affirmed the role of prophylactic antibiotics in managing endodontic pain. In a randomized controlled study titled *The Role of Prophylactic Antibiotics in the Management of Postoperative Endodontic Pain*, researchers focused on the effects of prophylactic antibiotic usage. The study divided patients into two groups: Group A consisted of 65 patients who received a 400 mg tablet of ibuprofen before the procedure, followed by one tablet every eight hours on the first day, and one tablet as needed for pain. Group B, comprising 64 patients, followed the same ibuprofen regimen as Group A. However, they also received amoxicillin and clavulanic acid (one tablet before the procedure and then one tablet twice daily for three days). The patients recorded their pain intensity at eight-hour intervals using the VAS and noted the total number of ibuprofen tablets used. The study concluded that prescribing antibiotics to manage post-endodontic treatment pain led to lower pain levels and reduced ibuprofen consumption. This finding is particularly relevant in cases where NSAIDs are contraindicated, as short-term antibiotic use proved effective in alleviating pain [20]. While these results are promising, there are several limitations and potential biases to consider. First, the sample size, though relatively large, may not fully represent the broader population due to potential selection bias. The study's reliance on self-reported pain scales introduces subjective variance, which may not accurately reflect the objective severity of pain. Additionally, the study did not account for individual variations in pain tolerance or the placebo effect, which could significantly influence self-reported pain levels. The lack of a placebo control group is a notable limitation, as it makes it challenging to differentiate the actual effect of antibiotics from psychological factors. Furthermore, the study's short duration does not address the potential long-term implications of antibiotic use, such as the development of antibiotic resistance. These considerations suggest that while the study offers valuable insights into the efficacy of antibiotics for managing PEP.

 *Analgesic and Antibiotic Combination*

Antibiotics and analgesics are crucial in controlling dental infections and pain. However, several global studies have reported their overprescription [32]. Currently, there is a significant discussion about the local delivery of antibiotics in theory and clinical practice. This debate becomes particularly relevant during mechanical instrumentation in endodontics, as some parts of the root canal may remain unprepared, enabling the infection to persist. To achieve the best possible results, the use of intracanal medicaments, including antibiotics, is recommended. A review by Mittal and Jain on using antibiotics as an intracanal medicament in endodontics concluded that local application of antibiotics within the root canal system could be more effective than systemic administration [33]. This approach is further supported by other studies advocating the combined use of corticosteroids and antibiotics as intracanal medicaments, which have shown positive results [34]. Additional studies have been conducted to identify the most effective intracanal medicament following RCT in patients with pulpal necrosis and acute apical periodontitis. These studies categorized patients into three groups: Group 1 received Ledermix paste, Group 2 was treated with calcium hydroxide paste, and Group 3 received no dressing. The results showed that Ledermix paste was the most effective in reducing postoperative pain, with a rapid onset of action. However, it is important to consider the study's methodology, such as the sample size and the criteria for patient selection, as these factors could influence the outcomes. Additionally, the long-term effects of these medicaments were not explored, which is crucial for understanding their overall efficacy and safety [35].

Anesthesia

The American Association of Endodontics has highlighted the challenges of achieving effective anesthesia in patients already experiencing pain. These challenges are attributed to several factors, including the excitability of nociceptors, decreased excitability thresholds, TTX receptors, altered resting potentials, and the patient's level of fear and anxiety. A recent article explored various methods to enhance the clinical success of local anesthesia in patients with symptomatic irreversible pulpitis and pain management. These methods include the Gow-Gates technique, using buffered anesthetic solutions, supplemental buccal infiltration with articaine, the effect of preemptive medications on success, and proven supplemental methods such as intraosseous anesthesia, intraligamentary (PDL) injection, nitrous oxide, intrapulpal anesthesia, anesthesia techniques for maxillary molars in patients with symptomatic irreversible pulpitis, incision and drainage (I&D) procedures, and new formulations like Kovanaze Nasal Spray and EXPAREL (liposomal bupivacaine) [36]. Local anesthetics are commonly used alone or in combination with oral medication for pre-procedural or background pain management in endodontic treatments. A recent study that compared different interventions and outcomes found that the most effective interventions in the mandible were premedication with diclofenac sodium and paracetamol followed by IANB or IANB with 2% lidocaine coupled with buccal infiltration of 4% articaine. Both interventions showed greater efficacy compared to the control group. An indirect comparison indicated that premedication with ibuprofen and paracetamol before IANB was the most effective intervention compared to control in the mandible. However, no significant differences were found between interventions in the maxilla [37].

Premedication

Patients with a low pain threshold often benefit from pretreatment with analgesic drugs or supplementary analgesic injections. These are used in conjunction with local anesthesia to enhance the anesthetic efficacy of 2% lidocaine, especially when given as an IANB [38]. In these cases, anti-inflammatory drugs are commonly used [39]. A study was conducted to evaluate the analgesic effects of three different drugs (ketorolac, tapentadol, and etodolac) when used as premedication for managing endodontic pain. The study found that a single oral dose of 10 mg ketorolac or 100 mg tapentadol, administered 30 minutes before endodontic treatment, significantly reduced postoperative pain compared to 400 mg etodolac [19]. This approach also aimed to increase the efficiency of pulpal anesthesia [40].

Valacyclovir

Valacyclovir, an antiviral drug, has shown promise in treating acute apical abscesses in endodontics. A study investigating valacyclovir combined with amoxicillin in managing pain involved 20 patients with moderate-to-severe pain. They were randomly assigned to receive either the medication combination or a placebo. The study found a significant decrease in pain in the valacyclovir group [41]. However, the study's small sample size and reliance on self-reported pain measures may limit the generalizability of these results. While effective in the short term, additional research with a larger cohort is needed to confirm these findings [41]. Table 1 shows a summary of pain management strategies in endodontics.

**Table 1:** Summary of pain management strategies in endodontics: behavioral, pharmacological, and anesthetic approaches. NSAIDs, nonsteroidal anti-inflammatory drugs

Aspect	Details
Behavioral management	Managing patient anxiety and expectations through environmental adjustments, effective communication, distraction methods, and rapport-building. High dental fear often develops in early childhood.
Pharmacological management	
Nonopioid analgesics	Include acetaminophen. Used for pain management in various dental conditions
Opioid analgesics	Such as tramadol and codeine. Reserved for severe cases with careful consideration due to the potential for addiction and adverse effects
NSAIDs	Include ibuprofen, flurbiprofen, ketorolac tromethamine, etodolac, tenoxicam, and naproxen. Effective for relieving post-endodontic pain. Diclofenac sodium is particularly used for pain and swelling postoperatively
Corticosteroids	Known for their anti-inflammatory and immune-suppressive effects. Beneficial for managing endodontic pain
Trypsin-chymotrypsin	Used to reduce postoperative swelling and manage severe pain
Opioids/narcotic analgesics	Reserved for severe cases where nonnarcotic analgesics are insufficient
Prophylactic antibiotics	Used to manage postoperative endodontic pain, especially when NSAIDs are contraindicated
Analgesic and antibiotic combination	Local application of antibiotics within the root canal system can be effective
Anesthesia	Various methods explored to enhance clinical success in patients with symptomatic irreversible pulpitis and pain management
Premedication	Use of analgesic drugs or supplementary analgesic injections before treatment to enhance anesthetic efficacy
Valacyclovir	Used as an adjunct for treating acute apical abscesses, effective in combination with amoxicillin for pain suppression

Clinical management

During RCT, patients may experience acute exacerbations or flare-ups, which can cause significant distress and require an unexpected clinic visit. Therefore, managing interappointment pain is crucial. The etiology of this pain is complex, involving microbial factors (a disturbed host-bacteria relationship), chemical injury, or mechanical injury to pulpal or periradicular tissues [42].

Incision and Drainage

I&D is performed to evacuate pus, microorganisms, and toxic products from the periradicular tissue, which helps decompress the tissue and alleviates pain. In cases where an abscess occurs after the obturation of the root canal system, incising the fluctuant tissue may be the only viable emergency treatment, especially if the root canal filling is deemed adequate [42]. A study involving 81 patients with endodontic emergencies compared actual I&D (I&D with a mock I&D procedure following endodontic debridement). Patients were randomly assigned to either real or mock I&D, and all received pain medication postprocedure. Surprisingly, the mock I&D group reported higher success in pain reduction without opioid use compared to the actual I&D group, suggesting that patient perception of intervention might significantly influence pain management outcomes in endodontic emergencies [43].

Re-instrumentation

Re-instrumentation, or reentering the tooth, is a critical step in the definitive treatment of a symptomatic tooth. This process involves opening the tooth for access opening, cleaning, shaping, providing drainage, and performing copious irrigation. These steps are essential to remove residual microbial debris that could otherwise cause further postoperative symptoms [42]. In two distinct cases, effective drainage played a crucial role in managing endodontic emergencies. The first case involved a 22-year-old male with severe palatal swelling and pain related to nonvital teeth. Drainage was achieved through an incision and apical trephination, leading to rapid symptom resolution. The second case featured a 53-year-old female with intense jaw pain due to a periapical radiolucency under a fixed dental prosthesis. Here, the removal of the prosthesis and subsequent drainage through the affected tooth provided immediate pain relief. In both instances, multi-visit RCTs with triple antibiotic paste were successfully employed following the drainage procedures [44].

Trephination

Trephination is a specific endodontic procedure that involves creating an opening in the alveolar cortical plate or the apical foramen. The primary purpose of this procedure is to perforate the area and drain the inflammatory exudate. Trephination has been proven to provide prompt relief from pain. Whether or not antibiotic coverage is needed in conjunction with this procedure depends on the patient's overall health status. Following trephination, there is generally minimal need for analgesics [45]. In a study with 11 patients, guided endodontic surgeries using a trephine effectively treated periapical lesions on 13 teeth. The trephine was instrumental for precise osteotomy, crucial in resecting the apical portions of the roots. All surgeries achieved their objectives with high accuracy, as indicated by minimal deviations from the planned procedures [46]. However, it's important to consider that the small sample size and the focus on a specific surgical technique may limit the generalizability of these findings. Further research with a larger patient cohort is needed to fully validate the efficacy and applicability of trephine use in varied endodontic conditions.

Occlusal Reduction

The effectiveness of occlusal reduction in endodontics remains a topic of debate. A systematic review and meta-analysis conducted by Shamszadeh et al., which included six randomized controlled trials with 344 participants, concluded that occlusal reduction did not significantly reduce postoperative pain during the first two days following the procedure. However, positive results were noted on the third day for controlling PEP [47]. In a trial with 308 patients, the effect of occlusal reduction on post-treatment pain in mandibular posterior teeth with symptomatic irreversible pulpitis was assessed. Patients were randomized into groups with and without occlusal reduction, undergoing two-visit RCTs. Results indicated that occlusal reduction significantly reduced pain at 12 and 24 hours post-instrumentation, decreasing moderate-to-severe pain risk by 40% at 12 hours [48].

Platelet-Rich Fibrin (PRF)

PRF is a living biomaterial consisting of a fibrin matrix, platelet cytokines, growth factors, and cells. It has traditionally been used in oral and maxillofacial management to promote healing. Its application in endodontic surgeries has also been shown to be effective in pain management. A study titled *Evaluation of Hydroxyapatite Granules, CERAMENT™, and Platelet-Rich Fibrin in the Management of Endodontic Apical Surgery* found that PRF was superior to hydroxyapatite and CERAMENT in reducing pain, suggesting its potential as a recommended option in endodontic pain management [49].

Cryotherapy

Cryotherapy, recognized for its therapeutic use of low temperatures, has also been adopted in dentistry. Vera et al. pioneered the application of cryotherapy in root canal procedures. They used a saline solution chilled to 2.5 °C and applied for five minutes using EndoVac, a specialized negative pressure device designed for root canal irrigation. This method aimed to reduce the root surface temperature, taking advantage of the known benefits of cold therapy in treating traumatic injuries and facilitating recovery in various surgical contexts [50]. The findings from this study indicated that this application of 2.5 °C cold saline as the final irrigant significantly reduces postoperative pain compared with a standard control group. Cryotherapy is thus an effective, economical, and safe method for alleviating postoperative discomfort in procedures involving single-visit RCTs [51]. Table 2 shows effective pain management techniques in endodontic treatments.

**Table 2:** Effective pain management techniques in endodontic treatments.

Clinical management techniques	Effectiveness
Incision and drainage	Alleviates pain by evacuating pus and toxic products, especially useful in cases of abscess post-obturation
Re-instrumentation	Essential for removing residual microbial debris, preventing postoperative symptoms
Trephination	Offers prompt pain relief by draining inflammatory exudate
Occlusal reduction	Effective in controlling post-endodontic pain, especially from the third day posttreatment
Platelet-rich fibrin (PRF)	Reduces pain effectively in endodontic surgeries
Cryotherapy	Significantly reduces postoperative pain in single-visit root canal treatments

Future prospects

GABAergic Signaling in Modulation of Dental Pain

γ-Aminobutyric acid (GABA) is an inhibitory neurotransmitter that is present in high levels in inflamed human dental pulp. Several studies have investigated the expression of GABA and its receptors in dental pulp and its role in dental pain transmission [12]. One study hypothesized that dental pulp contains functional GABA receptors using a GTPγ35S binding assay. This finding drew further attention to the potential of this system in initiating and managing endodontic pain. The study stated the following: "Because GABA agonists are antihyperalgesic in other tissues, GABA agonists may have similar effects in dental pulp, assuming that this tissue contains GABA receptors" [52]. These research studies have highlighted a promising modality for managing pain during endodontic procedures, and there is considerable potential for future applications. However, challenges such as limited clinical data and the need for specific targeting of GABA receptors to avoid side effects must be addressed. Further research is essential to establish the practicality and long-term efficacy of GABAergic treatments in dental practice.

Acupuncture

Acupuncture has been widely used for a long time in China to mitigate pain and promote healing [14]. Additionally, it has often been considered as a treatment for post-extraction pain [53]. Several studies have been conducted in dentistry to explore the efficacy of acupuncture, particularly in endodontics, for postoperative pain relief. In one randomized controlled trial, preoperative acupuncture was studied as a means to manage or alleviate pain caused by symptomatic apical periodontitis. The results favored the intended aim, showing more significant pain reduction in the acupuncture group compared to the placebo group at all intervals throughout the day [54]. Another study compared the efficacy of acupuncture with that of ibuprofen and found that acupuncture was a safer option for the management of postoperative pain [53]. Acupuncture provides a nonpharmacological approach to treatment, and its role in endodontics is being further studied due to its potential benefits. A few studies have explored its adjunctive role with local anesthesia and reported that it can improve the efficacy of local anesthesia when administered as a nerve block. It also helped calm the patients, reducing their apprehension and minimizing the need for oral analgesics, which can lead to other side effects [55]. Despite the promising results of acupuncture in managing endodontic pain, there are notable challenges and limitations to its wider adoption in dental practice. One significant challenge is the variability in individual responses to acupuncture, making it difficult to predict efficacy across different patients. Additionally, the need for practitioners to have specialized training in acupuncture limits its availability in general dental settings. The underlying mechanisms of how acupuncture specifically affects dental pain are not fully understood, posing a challenge to integrating it into evidence-based dental practices. There is also a scarcity of large-scale, long-term studies that conclusively establish the benefits and potential risks of acupuncture in endodontics. Therefore, while acupuncture presents as a viable non-pharmacologic option for pain management, its application in endodontics needs more robust clinical evidence to overcome these challenges and gain wider acceptance.

In Silico Modulation

Researchers are constantly exploring new ways to alleviate the pain associated with moderate-to-severe endodontic pulpitis. One such study focused on inhibiting interleukin-8 (IL-8) found in inflamed periapical tissues. The goal was to use a small molecule, ZINC14613097, to inhibit IL-8 and develop a drug that could provide therapeutic effects against endodontic pain. This molecule also holds the potential to serve as an inhibitor of IL-8, which could have clinical implications in the future [56]. While the in silico approach of using small molecules like ZINC14613097 to inhibit IL-8 presents a novel pathway for endodontic pain relief, it faces several challenges and limitations. One of the primary challenges is translating these in silico findings into effective clinical treatments. The process of drug development from computational modeling to clinical application is complex and lengthy, often encountering unforeseen obstacles in efficacy and safety in human trials. Additionally, the specificity of such molecules to target IL-8 without affecting other crucial biological processes poses a significant challenge. There is also the potential for unforeseen side effects, which requires extensive clinical trials to ensure safety. Another limitation is the current lack of comprehensive in vivo data to support these findings, which is crucial for understanding the real-world effectiveness of these treatments. Therefore, while this approach is promising, substantial further research is required to navigate these challenges and fully realize the potential of small molecule inhibitors in managing endodontic pain.

Low-Level Laser Therapy

During root canal procedures, laser photonic energy can be a helpful addition to the optimal treatment modalities. Additionally, there is a growing interest in exploring the potential of photobiomodulation for pain modulation in this field [57]. To establish the definitive role of low-level laser therapy (LLLT) in pain management, it is crucial to gather a significant number of case reports treated with laser therapy. However, it is essential to note that some side effects associated with lasers, including skin hyperpigmentation, erythema, eye injury, and thermal injury, limit their use to professionally trained dentists [58]. Furthermore, a systematic review of randomized controlled clinical trials has concluded that the effectiveness of phototherapy in reducing PEP after RCT remains debatable. To better understand the true potential for success, more randomized controlled trials involving laser interventions need to be conducted following established guidelines and parameters [54-55]. There is a glimmer of hope in research that suggests lasers have been therapeutically used for various applications, including direct irradiation of root canals, adjunctive use with irrigants in combination with photosensitizers for antimicrobial photodynamic therapy, and pain management through photobiomodulation [57]. However, it is worth noting that only a limited number of studies and researchers have reported significantly less postoperative pain with LLLT. Notably, one study confirmed that LLLT can be a viable alternative for managing postoperative pain [59]. Table 3 shows emerging techniques in endodontic pain management.

**Table 3:** Emerging techniques in endodontic pain management: future perspectives IL-8, interleukin-8

Future prospect	Description and potential
GABAergic signaling	γ-Aminobutyric acid (GABA) is an inhibitory neurotransmitter in the inflamed dental pulp. Research suggests the presence of functional GABA receptors in dental pulp, offering the potential for managing endodontic pain.
Acupuncture	Used for pain mitigation and healing. Studies show it can be effective for postoperative pain relief in endodontics, with benefits over ibuprofen. It's being explored as an adjunctive therapy with local anesthesia, reducing apprehension and minimizing the need for oral analgesics.
In silico modulation	Focuses on inhibiting IL-8 found in inflamed periapical tissues. The use of small molecules like ZINC14613097 is being researched to develop drugs that could provide therapeutic effects against endodontic pain.
Low-level laser therapy (LLLT)	Laser photonic energy is used in root canal procedures and photobiomodulation for pain modulation. Effectiveness in reducing post-endodontic pain remains debated, with more randomized controlled trials needed. Some side effects like skin hyperpigmentation and thermal injury limit its use. A few studies report reduced postoperative pain with LLLT, suggesting it as a viable alternative for pain management.

## Conclusions

This narrative review has provided a succinct overview of the multifaceted approaches to pain management in endodontics, emphasizing both traditional and innovative methods. We've examined pharmacological strategies like analgesics, antibiotics, and corticosteroids, alongside emerging treatments such as PRF, cryotherapy, GABAergic signaling, acupuncture, in silico modulation, and LLLT. These diverse techniques underscore a shift toward holistic, patient-centered care, reflecting the complexity of dental pain and the need for personalized treatment strategies. As endodontics continues to evolve, ongoing research and clinical trials are crucial to validate and refine these pain management methods, aiming to enhance patient care in this dynamic field.
